# The Moderating Effects of Physical Activity on the Relationships between Child Maltreatment and Health Outcomes among Korean Adolescents: A Secondary Analysis of the 2020 Korean Children and Youth Rights Survey

**DOI:** 10.3390/jcm12144574

**Published:** 2023-07-09

**Authors:** Suryeon Ryu, Zan Gao

**Affiliations:** School of Kinesiology, University of Minnesota—Twin Cities, Minneapolis, MN 55455, USA; gaoz@umn.edu

**Keywords:** adverse childhood experiences, depression, emotional abuse, physical activity, mental health, self-esteem

## Abstract

Adverse childhood events (ACEs) are associated with poor health outcomes and behaviors. Research has shown that physical activity (PA) can have a protective effect on the relationship between ACEs and health outcomes. This study aimed to explore the moderating effects of PA on the relationships between ACEs (physical and emotional abuse) and physical and psychological health (depression and self-esteem) among South Korean middle school students. The data used in this study were from the 2020 Korean Children and Youth Rights Survey (KCYRS), which had 2640 participants. The results indicated that PA could lessen the negative effects of emotional abuse on physical health and self-esteem. However, there was no significant moderating effect of PA in the relationship between physical abuse and health outcomes. The findings suggest that, while PA might be a protective factor for individuals exposed to emotional abuse, the effects of abuse on engaging in PA and the benefits of PA can be complex. Further research is needed to understand how different types of ACEs affect individuals and how PA can mitigate negative impacts. Health professionals, educators, and stakeholders should provide more opportunities for PA to support healthy behaviors and prevent negative health outcomes in adulthood for youth exposed to ACEs.

## 1. Introduction

Adverse childhood experiences (ACEs) are traumatic events that children between the ages of 0 to 17 may encounter [1]. This term encompasses all forms of violence, abuse, or neglect, as well as problems related to substance abuse, mental health issues, parental separation, suicide, death, or incarceration of a family member, which can significantly impact a child’s safety and stability [1]. Regrettably, ACEs are widespread in our society. In 23 states in the United States, approximately 62% of adults have reported experiencing at least one ACE during their early life [2]. Similarly, in South Korea, the incidence of ACEs has continuously increased each year, with 37,605 cases being reported in 2021 [3]. Notably, 42.6% of cases involved more than one ACE, and the prevalence of emotional abuse, physical abuse, and neglect was 32.8%, 15.4%, and 7.4%, respectively. It is also noteworthy that children with physical and emotional abuse experiences were most prevalent among those who had experienced multiple ACEs.

Early life adversities raise public health concerns due to the growing evidence that demonstrates the relationship between ACEs and poor health in adolescence and adulthood [4]. When children experience one of the ACEs, they are more likely to face negative health outcomes, including mental health issues (e.g., depression, low self-esteem), physical limitations, various chronic illnesses (e.g., cancer, heart disease), and an increased risk of premature death [4,5,6,7,8]. Moreover, ACEs can trigger stress responses that interfere with a child’s cognitive and emotional development, and stress-response nerve system, which are closely linked to academic achievement and social relationships [9]. Specifically, ACEs may have a negative impact on a child’s social relationships and ability to adapt, potentially leading to social isolation, anger, and hostility [4]. Additionally, research indicates that ACEs are linked to unhealthy behaviors such as physical inactivity, substance use, unhealthy diet, smoking, alcohol abuse, violence, and sleep disturbance [4,6,8]. Thus, exposure to ACEs can potentially have a direct or indirect harmful impact on a child’s healthy development and overall well-being, resulting in a low health-related quality of life (QoL) and the adoption of risky health behaviors in adulthood [10,11,12]. These findings highlight the concerning prevalence of ACEs and their potentially severe impact on children’s health and well-being.

It is crucial to recognize that disadvantaged children are at a heightened risk of experiencing such adversities. A previous study conducted in the United States revealed that individuals with lower levels of income and education were more susceptible to experiencing higher levels of ACEs compared to those in higher income and education brackets [2,13]. Similarly, the 2020 Review of Korean Children’s and Youth Rights highlighted that children and adolescents from low-income backgrounds had a greater prevalence of child maltreatment (e.g., emotional abuse, physical abuse) compared to their counterparts [14]. The unequal distribution of childhood adversity is a concerning issue, as it can further widen existing health disparities. This is because the adverse health outcomes resulting from ACEs can negatively affect the social determinants of health, including educational attainment, career prospects, and socioeconomic status. Consequently, this can contribute to health and socioeconomic inequalities later in life [2,15]. Additionally, it is likely that unhealthy behaviors and stress generated from ACEs can be passed on to the next generation, further worsening health disparities in our society [1]. In summary, recognizing the significance of reducing health disparities highlights the necessity of developing evidence-based approaches to prevent ACEs within family settings, mitigating the negative health effects throughout one’s lifetime, and fostering equitable health outcomes.

Physical activity (PA) has been acknowledged as a cost-effective and non-pharmacological approach to improving an individual’s health in multiple ways. Research has consistently shown that daily PA has positive effects on various health outcomes in children and adolescents. These benefits include improvements in fitness and weight status, as well as a lower risk of chronic diseases [16]. Moreover, regular PA is widely recognized as a crucial lifestyle behavior that can reduce the likelihood of experiencing anxiety and depression while promoting psychological well-being [16,17,18,19,20]. Several studies have examined the protective effect of PA in alleviating the negative impact of ACEs on mental health [21,22,23]. These studies have demonstrated that PA participation plays a protective role in reducing the harmful effects of ACEs on stress, depression, and anxiety [12,21,22,23]. For instance, recent research has indicated that PA can mitigate the negative impact of ACEs and depression on an individual’s functional dependence, including their ability to engage in daily and leisure activities. These findings underscore the importance of incorporating PA into one’s lifestyle throughout their lifetime [22].

Previous studies have suggested that PA can serve as a promising strategy to improve overall health for individuals who have experienced ACEs. However, there remains a lack of sufficient and consistent evidence to fully eliminate uncertainty surrounding the relationship between PA and ACEs, as well as the extent to which PA mitigates the link between ACEs and physical and mental health. Notably, to the best of our knowledge, no preceding studies have explored the protective nature of PA on health outcomes among South Korean adolescents. Adolescence is a crucial life stage of human development, characterized by accelerated psychological and biological changes [24]. During the transition from adolescence to adulthood, individuals often develop similar lifestyle habits, including PA patterns [25]. In South Korea, education is mandatory until middle school and, upon graduation, Korean middle-schoolers experience a significant change in their educational pathways. It is not uncommon for high school dropout rates to be higher than middle school dropout rates among Korean adolescents [26,27]. Recent research suggests that ACEs and disengagement from school can increase the likelihood of delinquent behavior among young individuals who do not have the protective factor of school education [28]. Thus, it is crucial to carefully examine effective strategies that can mitigate the impact of adverse experiences during early adolescence, as they can potentially influence their immediate trajectory and may also lead to negative health outcomes in the future.

The primary aim of this study is to investigate the moderating role of PA in the association between ACEs and physical and psychological health in middle-schoolers from South Korea. Specifically, this study focuses on physical abuse and emotional abuse (verbal abuse), which are commonly recognized types of child maltreatment and reported to be the most frequent types of ACEs experienced by South Korean adolescents who have experienced at least one ACE [3,9]. Using secondary analysis, this study utilized data from the 2020 Korean Children and Youth Rights Survey (KCYRS) to examine the moderating effect of PA on the relationship between ACEs (specifically physical abuse and emotional abuse) and three outcome variables: sense of physical health, self-esteem, and depression. Additionally, gender, education level, economic status, academic achievement, and family structure were included as covariates. The research hypotheses were that PA would weaken the positive association between ACEs and (1) physical health, (2) self-esteem, and (3) depression.

The findings of this study can fill the knowledge gap regarding the health benefits of PA in adolescents who have experienced physical and emotional abuse. Moreover, these findings have the potential to contribute to improvements in health disparities among this population. Additionally, this study provides valuable insights within the context of the Coronavirus Disease 2019 (COVID-19) pandemic, considering the impact of lockdown policies and school closures. These circumstances have led to increased time spent at home, potentially heightening the risk of child abuse and increasing the likelihood of youths who already experience abuse facing further maltreatment.

## 2. Materials and Methods

### 2.1. Data and Participants

This study is a secondary analysis of publicly available data from the 2020 KCYRS, which is conducted annually by the National Youth Policy Institute (NYPI) in South Korea [14]. The data was collected between July and October 2020, and the survey responses provided by the students were de-identified to ensure anonymity. The KCYRS is a nationwide, cross-sectional survey designed to investigate and monitor the human rights of Korean children and adolescents [14]. In order to obtain survey responses from a nationally representative sample of school-aged children and adolescents, the KCYRS utilizes a stratified multistage clustering sampling design. The final sample for the 2020 KCYRS data collection included elementary (grades 4–6; *n* = 2896), middle (grades 7–9; *n* = 2704), and high school students (grades 10–12; *n* = 3023), resulting in a total of 8623 children and adolescents.

**Figure 1:**
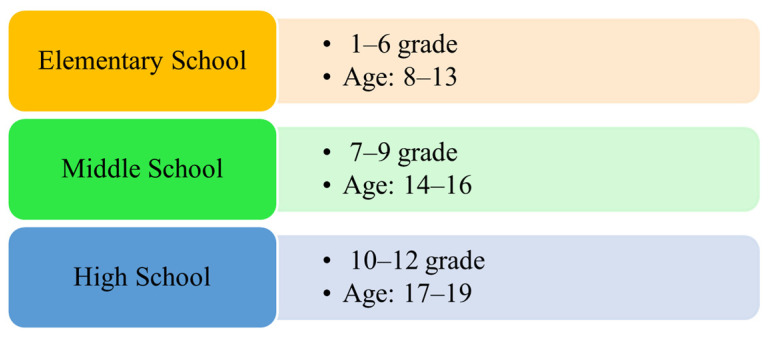
South Korea public education hierarchical levels.

In this study, we analyzed survey responses from middle school students in grades 7 to 9, which correspond to grades 9 to 11 in the United States (Figure 1). This age range represents the early stage of adolescence, and investigating their experiences and habits during this period is crucial as it can have implications for their later stage adolescence. It is important to note that South Korea has various types of high school with different learning objectives, such as general, autonomous private (with an individualized education curriculum), and specialized types depending on majors (e.g., science, language, arts), as well as employment-focused types. These differences in high school education from middle school education led to the exclusion of high school students from this study, as failing to account for these differences may lead to distorted associations in the analysis. Additionally, individuals with incomplete data related to the exposures, outcomes, moderators, and covariates were excluded. As a result, the final sample size for statistical analyses consisted of 2640 participants.

### 2.2. Measures

The survey items of KCYRS have been developed based on the articles of the United Nations Convention on the Rights of the Child (UNCRC) and were primarily created by researchers at NYPI. In order to ensure the reliability and validity of the KCYRS, experts from various fields are recruited by the NYPI to review and revise the survey items annually, taking into account the findings from previous years. These modified survey items are approved by Statistics Korea (approval number: 402001) to ensure their accuracy and effectiveness [14].

#### 2.2.1. Predictor: Child Maltreatment

The exposure variables include physical abuse (physical punishment) and emotional abuse (verbal abuse). Students self-reported the frequency of each type of child maltreatment using a 5-point Likert-type scale (1 = none; 2 = once or twice per year; 3 = once or twice per two, three months; 4 = once or twice per month; 5 = once or twice per week) [14]. For physical and emotional abuse, participants were asked to report how often they experienced physical abuse and verbal abuse from their parents or legal guardians. Higher scores on the scale indicated a greater level of exposure to physical and emotional abuse, respectively.

#### 2.2.2. Outcome Variables

The outcome variables were self-perception of physical health, self-esteem, and depression. For physical health, students rated their health using a single item on a 4-point Likert scale, ranging from 1 (not very good) to 4 (very good) [14]. A higher score indicated a better perception of physical health. 

For self-esteem and depression, students self-reported three items each, using a 4-point Likert scale, ranging from 1 (strongly disagree) to 4 (strongly agree). The average of each score was used as the outcome measure. The three items used to assess self-esteem were selected from Rosenberg’s (1965) self-esteem scale [29]: (1) I feel that I’m a person of worth; (2) I feel that I have number of good qualities; and (3) I feel I do not have much to be proud of (reverse scored). For depression, the three items included: (1) I feel lonely for no reason; (2) I feel nervous for no reason; and (3) I feel sad and blue for no reason. Higher scores indicated better self-esteem and more severe depression. The Cronbach’s alpha values of self-esteem and depression scales were 0.79 and 0.89, respectively.

#### 2.2.3. Moderator: Physical Activity

PA was assessed with one item asking exercise frequency outside of school physical education (PE). The students rated their PA behavior based on a 4-point Likert scale (1 = none; 2 = once or twice per month; 3 = once or twice per week; 4 = at least three times per week) [14]. A higher score indicated that the student engaged in PA more frequently during their leisure time. Due to the data collection period coinciding with the COVID-19 pandemic, when all school lessons were delivered remotely, students were unable to fully participate in quality PE at home. Therefore, measuring leisure PA during school closures serves as a meaningful proxy of adolescents’ active engagement in PA. 

#### 2.2.4. Covariates

This study included self-reported biological gender (i.e., boys or girls), education level (i.e., grade 7, 8, or 9), economic status (i.e., good, fair, or poor), academic achievement (i.e., good, fair, or poor), and family structure (i.e., nuclear, single parent, grandparent, or other) as covariates. The original response types for academic achievement and economic status were originally 5- and 7-point Likert scales, respectively. To address missing data or incomplete responses, we used reclassified variables provided by the NYPI, which categorized the variables into three levels (i.e., good, fair, or poor). For family structure, students responded with whom they are currently living.

### 2.3. Data Analysis

All data analyses were conducted using SPSS IBM Statistics (version 28; Chicago, IL, USA) and the data are presented as weighted samples to enhance the representativeness of the study findings. First, missing responses were screened prior to data analyses and Cronbach’s alpha coefficients were tested to observe the internal consistency of the survey items. Descriptive statistics were performed to describe the characteristics of the participants and Pearson’s correlation test was used to examine relationships among key variables. 

The primary analyses were conducted using the PROCESS macro (Model #1), a statistical tool for testing moderating effects, to analyze the data. The independent variables (exposures) were physical abuse and emotional abuse. Physical health, self-esteem, and depression were selected as the dependent variables. Specifically, multiple moderation models were tested to determine the moderating role of PA. All independent and moderating variables were mean-centered to eliminate the multicollinearity issue, and a bootstrapping method (5000 bootstrap samples) was adopted. In addition, the analyses adjusted for the covariates and each abuse type. To illustrate the relationships between the dependent and independent variables in relation to PA, we generated 2-way interaction plots. The significance level was set at 0.05 and a 95% confidence interval was utilized. When the confidence interval does not contain zero, it indicates a significant effect.

## 3. Results

### 3.1. Descriptive Statistics and Correlation Analysis

The descriptive characteristics of participants are shown in Table 1. Among the 2640 Korean adolescents who provided complete responses for key variables in this study, 1350 (51.7%) were males and 1290 (48.3%) were females. There was a total of 2640 participants in this study, with 929 (33.1%) in Grade 7, 850 (32.2%) in Grade 8, and 861 (34.8%) in Grade 9. The majority of adolescents came from nuclear families (90.7%) and reported a high household economic status (56.8%). In terms of academic achievement, 44.3% of adolescents reported moderate levels, 31.3% high levels, and 24.5% low levels. The mean (standard deviation; SD) score for PA participation was 2.58 (1.12), indicating the average frequency of exercise engagement was about 1–2 times per week during the pandemic when schools were shut down. In terms of physical and emotional abuse, adolescents reported mean (SD) scores of 1.33 (0.72) and 1.65 (1.11), respectively. These scores indicate that the average adolescent experienced emotional abuse more than physical abuse. The mean (SD) scores were 3.15 (0.64), 2.98 (0.70), and 1.88 (0.86) for perceptions of physical health, self-esteem, and depression.

Table 2 illustrates the correlations of the study variables in this study. The results show that all variables were significantly correlated with one another. In detail, exposures (i.e., physical abuse and emotional abuse) were positively correlated (r = 0.60, *p* < 0.01) with each. PA had positive correlations with physical health (r = 0.25, *p* < 0.01) and self-esteem (r = 0.19, *p* < 0.01), and negative correlations with physical abuse (r = −0.04, *p* < 0.05), emotional abuse (r = −0.05, *p* < 0.01), and depression (r = −0.12, *p* < 0.01). While both exposures had negative correlations with physical health and self-esteem, they had positive correlations with depression. Physical health was positively correlated with self-esteem (r = 0.32, *p* < 0.01), but physical health (r = −0.28, *p* < 0.01) and self-esteem (r = −0.36, *p* < 0.01) had a negative correlation with depression.

### 3.2. Moderation Analyses

**Figure 2:**
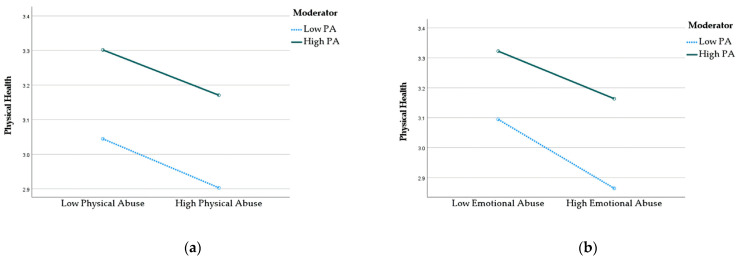
(**a**) Interaction effect between PA and physical abuse on physical health. (**b**) Interaction effect between PA and emotional abuse on physical health.

The moderation effects of PA on the connections between exposures (physical and emotional abuse) and physical health are presented in Table 3. Although the association between physical abuse and physical health did not exhibit a significant moderating effect of PA, a significant moderating effect of PA was observed in the connection between emotional abuse and physical health (B = 0.02, S.E. = 0.01, *p* < 0.05, 95% CI [0.00, 0.04]). Specifically, unstandardized beta coefficients for emotional abuse and interaction were −0.07 and 0.02, indicating that PA mitigates the negative impact of emotional abuse on physical health. The result shown in Figure 2 demonstrated that adolescents with a higher PA engagement reported better physical health even among adolescents exposed to higher emotional abuse. The plot shows that the lines are not parallel and the slope of the low PA is steeper than the high PA group, meaning PA attenuates the effect of emotional abuse on physical health. 

**Figure 3:**
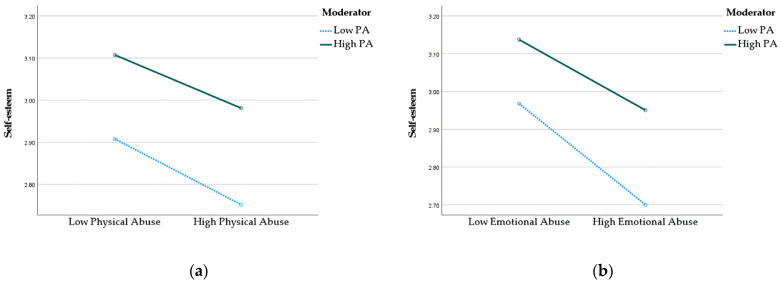
(**a**) Interaction effect between PA and physical abuse on self-esteem. (**b**) Interaction effect between PA and emotional abuse on self-esteem.

Next, the results of the moderation effects of PA on the relationships between exposures (i.e., physical and emotional abuse) and self-esteem are demonstrated in Table 4. It is indicated that PA significantly moderated the association between emotional abuse and self-esteem (B = 0.02, S.E. = 0.01, *p* < 0.05, 95% CI [0.00, 0.04]). The findings of Figure 3 indicate that adolescents who engage in PA more often tend to have better self-esteem. Moreover, the negative impact of emotional abuse on self-esteem is less pronounced among adolescents who engage in more PA, as opposed to those who engage in less PA. No significant moderating effect of PA was observed regarding the relationship between physical abuse and self-esteem (*p* = 0.76, 95% CI [−0.03, 0.04]). 

**Figure 4:**
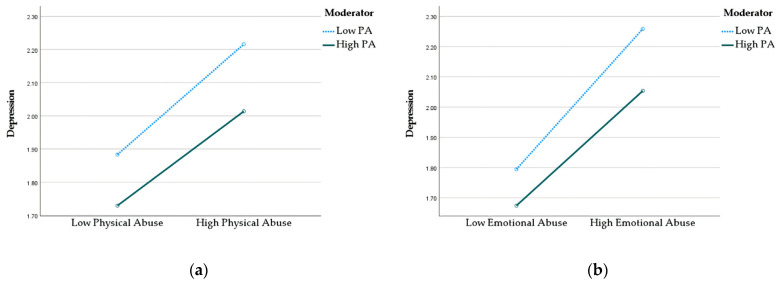
(**a**) Interaction effect between PA and physical abuse on depression. (**b**) Interaction effect between PA and emotional abuse on depression.

Lastly, Table 5 presents the results of moderating analyses of PA on the relationships between exposures (i.e., physical and emotional abuse) and depression. The results suggest that PA did not significantly moderate in any of the relationships, unlike the other dependent variable. However, the results in Figure 4 demonstrate that adolescents who engage in PA are likely to experience reduced feelings of depression. Furthermore, higher levels of PA have been found to mitigate the positive relationship between emotional abuse and depression.

## 4. Discussion

The prevalence of ACEs has been increasing in South Korea, with emotional and physical abuse being the most prevalent forms of harmful exposures that can result in long-term health disparities. PA has been identified as a beneficial approach for promoting healthier youth development and there is scientific evidence supporting its protective effects on overall health [16,30]. Nevertheless, there is a lack of research examining the associations between PA and ACEs, as well as the moderating effects of PA on the physical and mental health of South Korean adolescents. Therefore, this secondary analysis aimed to explore the moderating effects of PA on the relationships between ACEs (i.e., physical abuse and emotional abuse) and health outcomes (i.e., physical health perception, self-esteem, and depression).

Our findings indicate that adolescents exposed to higher levels of physical and emotional abuse reported significantly lower self-esteem, poorer physical health perception, and more severe depression. Moreover, higher levels of PA participation showed positive associations with better physical health perception and self-esteem, as well as negative associations with depression. These findings are in accordance with preceding literature, reflecting the negative impact of ACEs on an individual’s health and the beneficial aspects of PA on physical and mental health [4,5,6,23,31]. Additionally, considering the complex relationship between PA engagement and health outcomes, it is plausible that adolescents with higher self-esteem and fewer depressive symptoms may be more likely to participate in PA [32,33].

Our study partially supported the hypotheses that PA can moderate the relationships between child maltreatment (i.e., physical and emotional abuse) and health outcomes (i.e., physical health, self-esteem, and depression). The results indicated that as adolescents’ PA participation increased, they perceived better physical health even when experiencing higher levels of emotional abuse. Furthermore, the study found that PA reduced the negative impact of emotional abuse on self-esteem, suggesting that adolescents who experienced emotional abuse and participated in more frequent PA had better self-esteem compared to their less active counterparts. These findings suggest that PA can act as a protective factor and adolescents who have experienced emotional abuse (verbal abuse) may benefit from adopting a physically active lifestyle. On the other hand, the moderating effect of PA on the relationship between emotional abuse and depression was not statistically significant. Additionally, while our moderation analyses indicated that PA mitigates the negative impact of emotional abuse on certain health outcomes, such as physical health and self-esteem, none of the moderating effects of PA were significant among adolescents exposed to physical abuse. 

In summary, the results of this study are consistent with previous research that suggests PA plays a moderating role in the relationships between emotional abuse and physical health, as well as between emotional abuse and self-esteem. However, the current study did not observe significant moderating effects of PA with regards to physical abuse. These findings partially contrast previous studies that consistently presented the profound protective impact of PA on the health of individuals with a history of child maltreatment [12,23]. For instance, a recent study showed that participating in regular PA can alleviate the adverse effects of ACEs on an individual’s health-related quality of life, including both physical and mental health. This retrospective study highlights the potential of PA as a promising approach to enhance the well-being of individuals who have experienced ACEs [12]. Similarly, an observational study using longitudinal data indicated that adults who were exposed to ACEs but had participated in group exercises during adolescence were less likely to receive a diagnosis of depression compared to those who did not engage in group exercises [23]. 

The discrepant findings of this study may be attributed to variations in study designs, particularly in terms of measurements and participant characteristics. Previous studies did not specifically focus on individual types of ACEs, but instead utilized a questionnaire that combined multiple sub-domains of ACEs. This approach created gaps in understanding the specific mechanisms through which PA influences the relationships between different forms of child maltreatment and an individual’s health. Thus, future research should aim to explore the underlying pathways involved in different types of ACEs and how PA can contribute to mitigating their negative impacts on an individual’s health. Additionally, while this study aimed to examine the well-being of adolescents, the majority of preceding retrospective studies have identified the role of PA in the health outcomes of adults who have experienced ACEs. 

Furthermore, our observations of insignificant moderating effects of PA on the physical and mental health of adolescents, particularly in comparison to those who have experienced emotional abuse, suggest that the effects of abuse on a child’s capacity to participate in PA can be complex and multidimensional. While both physical and emotional abuse have adverse impacts, the severity and the type of abuse, as well as the individual child’s unique experiences and circumstances, can influence their ability to engage in PA differently. For example, adolescents who have experienced physical abuse may suffer from chronic pain and mobility impairment resulting from physical injuries or disabilities, which can restrict their ability to engage in PA [9,34]. In addition, those who have experienced physical abuse may have developed negative associations with PA due to past traumatic experiences [35]. These negative associations may lead them to avoid PA as a coping mechanism, making it an unappealing strategy for physically abused adolescents. In contrast, adolescents with a history of emotional abuse may struggle with mental health issues such as depression, anxiety, or low self-esteem, which are well-acknowledged health outcomes that can be improved through participation in PA [16,17]. Therefore, when addressing the well-being of adolescents who have experienced ACEs, it is crucial to consider their unique experiences and circumstances in order to provide appropriate support and resources that can help improve their overall health. 

The present study has several noteworthy strengths. This study is one of the pioneering research endeavors that investigates the protective mechanisms of PA in relation to the well-being of individuals who have experienced child maltreatment. This research holds significant importance as it enhances our comprehension of the moderating role of PA in various pathways through which each type of child abuse affects physical and mental health. However, further theory-based investigations are warranted to explore the underlying psychological mechanisms that link different forms of child maltreatment, participation in PA, and overall health. These findings will provide valuable information for developing effective and appropriate PA strategies to improve the physical and mental health of adolescents, enabling them to adopt healthier lifestyles in adulthood. Moreover, these findings can serve as a valuable resource for researchers, healthcare providers, and stakeholders to develop future health promotion research programs and establish effective policies aimed at enhancing the well-being of children and adolescents who have been exposed to maltreatment. 

Another strength of this study is the use of a rigorously sampled survey response from a representative sample of South Korean adolescents. The 2020 KCYRS implemented a stratified multistage clustering sampling design to collect survey responses from a nationally representative sample of school-aged children and adolescents. Additionally, the substantial sample size of the KCYRS, along with the weighted estimates of the study population, enhances the validity and reliability of the research findings. To the best of our knowledge, no previous research has investigated the same topic among South Korean adolescents, emphasizing the significance of the findings in advancing our understanding of the role of PA in promoting adolescent health and well-being. Furthermore, the study results have the potential to broaden the generalizability of earlier research by providing new evidence on the moderating effect of PA in alleviating the adverse effects of child abuse on health outcomes among South Korean adolescents. By bridging the knowledge gap in this field, the observations of this study may contribute to the development of more effective interventions and policies aimed at enhancing the health and well-being of adolescents with history of maltreatment, not only in South Korea but also in other countries facing similar challenges. 

Despite the strengths of this study, there are also several limitations that should be taken into account. Firstly, the use of a cross-sectional survey design in the current study introduces potential biases such as selection bias, recall bias, and response bias. Although measures were implemented to minimize these biases through the use of a stratified multistage clustering sampling design, weighted sample estimates, and adjustment for covariates, it is important to recognize that these biases may still be present. Additionally, as a one-time-point cross-sectional survey, the KCYRS may not capture long-term associations between child abuse and overall health. Moreover, it is important to note that the current study used data from the 2020 KCYRS, a period when schools were closed due to the pandemic. The assessment of adolescents’ PA was based on a single question asking about engagement in exercise besides school PA. This limitation should be considered when interpreting the study’s results, as there is a lack of specific information regarding their PA behaviors during the 2020 school year. To address this limitation, future studies should incorporate more precise measures of PA, enabling researchers to determine whether adolescents are meeting the recommended PA guidelines (i.e., at least 60 min of moderate-to-vigorous PA) [36,37]. For instance, the use of an international PA questionnaire (IPAQ) that captures an individual’s engagement in light, moderate, and vigorous PA in the last 7 days would provide a comprehensive assessment of the adolescents’ PA levels and patters. Alternatively, objective PA assessment using accelerometers or wearable fitness trackers can offer more accurate data on adolescents’ PA behaviors. Additionally, while the study focused on young individuals’ self-perception of their physical well-being, it is essential to include a more inclusive assessment of physical health. This could involve evaluating physical limitations or fitness levels to better understand the protective effect of PA on the physical health of adolescents exposed to abuse.

## 5. Conclusions

The present study’s observations shed light on the practical implications of understanding the moderating mechanisms of PA in relationships between child abuse and physical and mental health outcomes (i.e., self-esteem and depression). Overall, this study emphasizes the importance of promoting PA as a means of improving the health outcomes of adolescents with a history of maltreatment and highlights the need for further research in this area. These practical implications extend beyond South Korea and can inform the development of PA interventions and policies to enhance the health and well-being of adolescents with a history of maltreatment in other countries facing similar challenges. To improve the physical and mental health outcomes of child abuse victims, integrating PA promotion programs into existing support systems may be an effective strategy. It is crucial to note that, when addressing the well-being of adolescents who have experienced ACEs, the distinct types of child abuse and their psychological backgrounds must be considered as unique health exposures to provide appropriate PA interventions. Specifically, future research is needed to identify effective PA interventions specifically for physically abused children, with a focus on enhancing their self-regulation abilities.

## Figures and Tables

**Table 1:** Characteristics of Korean adolescents in middle school.

Variables		Descriptive
Gender	Boy	1350 (51.7)
	Girl	1290 (48.3)
Education level	Grade 7	929 (33.1)
	Grade 8	850 (32.2)
	Grade 9	861 (34.8)
Family structure	Nuclear	2388 (90.7)
	Single parent	191 (6.9)
	Grandparent	23 (0.9)
	Other	38 (1.5)
Economic status	High	1507 (56.8)
	Moderate	963 (36.9)
	Low	170 (6.3)
Academic achievement	High	839 (31.3)
	Moderate	1162 (44.3)
	Low	639 (24.5)
PA		2.58 ± 1.12
Physical abuse		1.33 ± 0.72
Emotional abuse		1.65 ± 1.11
Physical health		3.15 ± 0.64
Self-esteem		2.98 ± 0.70
Depression		1.88 ± 0.86

Note: Descriptive statistics are presented as unweighted number (weighted percentage) for categorical variables and weighted mean ± standard deviation for continuous variables.

**Table 2:** Correlation matrix for predictors, outcome variables, and moderator.

Variables	1	2	3	4	5	6
1. PA	1					
2. Physical abuse	−0.04 *	1				
3. Emotional abuse	−0.05 **	0.60 **	1			
4. Physical health	0.25 **	−0.08 **	−0.15 **	1		
5. Self-esteem	0.19 **	−0.10 **	−0.16 **	0.32 **	1	
6. Depression	−0.12 **	0.14 **	0.24 **	−0.28 **	−0.36 **	1

Note: * *p* < 0.05; ** *p* < 0.01

**Table 3:** Moderating effects of physical activity on the relationships between exposures and physical health.

	**B**	**S.E.**	**t**	***p***	**95% CI**
Constant	3.64	0.09	39.50	<0.01	3.46, 3.82
Physical abuse	0.01	0.02	0.32	0.75	−0.03, 0.05
PA	0.14 **	0.01	12.43	<0.01	0.12, 0.16
Physical abuse × PA	0.00	0.02	0.18	0.86	−0.03, 0.03
F = 37.112, R^2^ = 0.11, _adj_ R^2^ = 0.00					
	**B**	**S** **.** **E** **.**	**t**	***p***	**95%** **CI**
Constant	3.51	0.10	36.51	<0.01	3.32, 3.70
Emotional abuse	−0.07 **	0.01	−5.14	<0.01	−0.09, 0.04
PA	0.14 **	0.01	12.45	<0.01	0.12, 0.16
Emotional abuse × PA	0.02 *	0.01	2.14	<0.05	0.00, 0.04
F = 37.69, R^2^ = 0.11, _adj_ R^2^ > 0.00					

Note: * *p* < 0.05; ** *p* < 0.01; all moderation analyses were adjusted for covariates and each exposure; 95% CI denotes 95% confidential interval (lower bound, upper bound); B = unstandardized regression coefficient; SE = standard error.

**Table 4:** Moderating effects of physical activity on the relationships between exposures and self-esteem.

	**B**	**S.E.**	**t**	***p***	**95% CI**
Constant	4.10	0.10	42.03	<0.01	3.91, 4.30
Physical abuse	−0.01	0.02	−0.61	0.54	−0.06, 0.03
PA	0.09	0.01	7.71	<0.01	0.07, 0.11
Physical abuse × PA	0.00	0.02	0.30	0.76	−0.03, 0.04
F = 58.02, R^2^ = 0.17, _adj_ R^2^ = 0.00					
	**B**	**S.** **E.**	**t**	***p***	**95%** **CI**
Constant	3.99	0.10	39.09	<0.01	3.79, 4.19
Emotional abuse	−0.08	0.01	−5.64	<0.01	−0.11, −0.01
PA	0.09	0.01	7.71	<0.01	0.07, 0.11
Emotional abuse × PA	0.02	0.01	1.98	<0.05	0.00, 0.04
F = 58.53, R^2^ = 0.17, _adj_ R^2^ > 0.00					

**Table 5:** Moderating effects of physical activity on the relationships between exposures and depression.

	**B**	**S.E.**	**t**	***p***	**95% CI**
Constant	0.31	0.12	2.56	<0.05	0.07, 0.55
Physical abuse	0.01	0.03	0.33	0.74	−0.04, 0.06
PA	−0.01	0.01	−0.88	0.38	−0.04, 0.02
Physical abuse × PA	0.04	0.02	1.80	0.07	−0.00, 0.08
F = 55.63, R^2^ = 0.16, _adj_ R^2^ > 0.00					
	**B**	**S.** **E.**	**t**	***p***	**95%** **CI**
Constant	0.59	0.13	4.64	<0.01	0.34, 0.83
Emotional abuse	0.17	0.02	9.76	<0.01	0.14, 0.20
PA	−0.01	0.01	−0.96	0.34	−0.04, 0.01
Emotional abuse × PA	−0.00	0.01	−0.27	0.79	−0.03, 0.02
F = 55.21, R^2^ = 0.16, _adj_ R^2^ = 0.00					

## Data Availability

The data that support the findings of the study are publicly available on the website entitled Korean Children Adolescents Data Archive, operated by National Youth Policy Institute in South Korea.
